# Supplementary material for: Population structure discovery in meta-analyzed microbial communities and inflammatory bowel disease using MMUPHin
Source: Genome Biol. 2022 Oct 3;23:208. doi: 10.1186/s13059-022-02753-4 (PMC9531436; doi:10.1186/s13059-022-02753-4)
Supplement: Supplementary file 1 — Additional file 1: Supplemental Figures. [file 13059_2022_2753_MOESM1_ESM.docx]

# Supplemental Figures

#
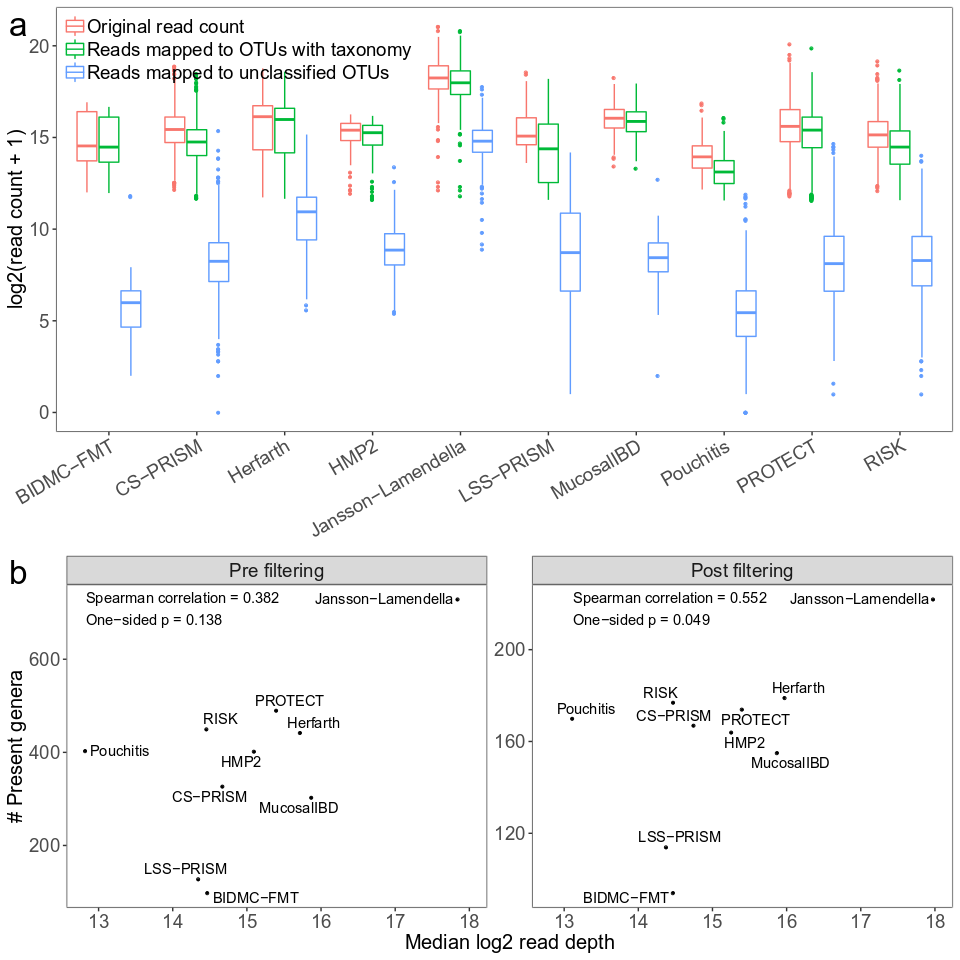


**Fig. S1: Read depth varies across studies and is correlated with number of detected taxonomic feature. a)** Per-sample total read depth varies across studies, and vast majority of reads are successfully classified with our bioinformatics protocols. **b)** Median read depth if each study is correlated with number of detected microbial features, both before and after prevalence and abundance filtering.


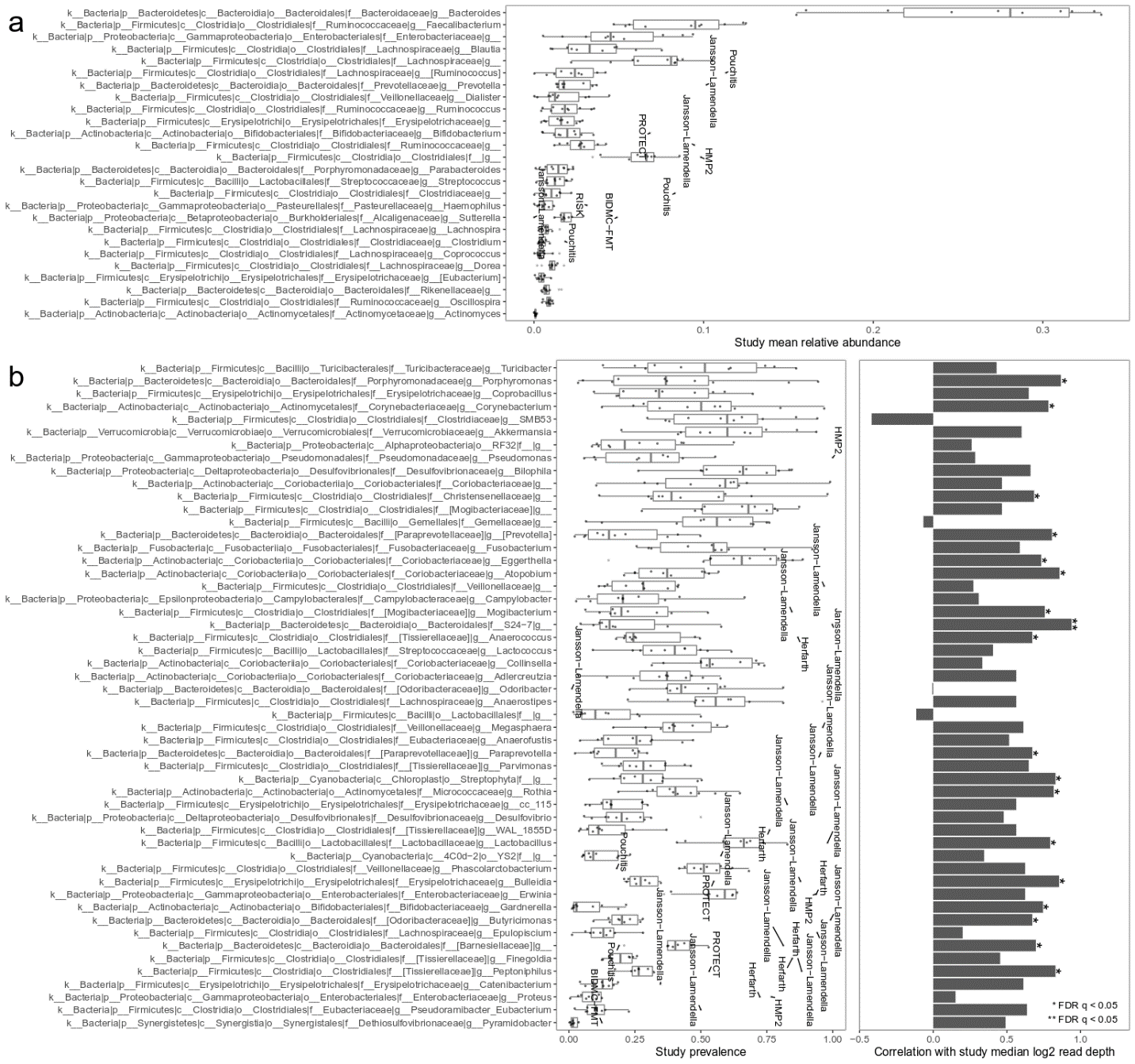


**Fig. S2: Globally prevalent taxa vary in study-specific mean abundance and prevalence, jointly affected by biological and technical factors**. **a)** For genera that are highly prevalent (>70% overall prevalence), per-study mean abundance can vary greatly. Each point represents a study-specific mean abundance of one feature. **b)** For other globally present genera, study-specific prevalence varied (left panel) and often correlated with median read depth (right panel).


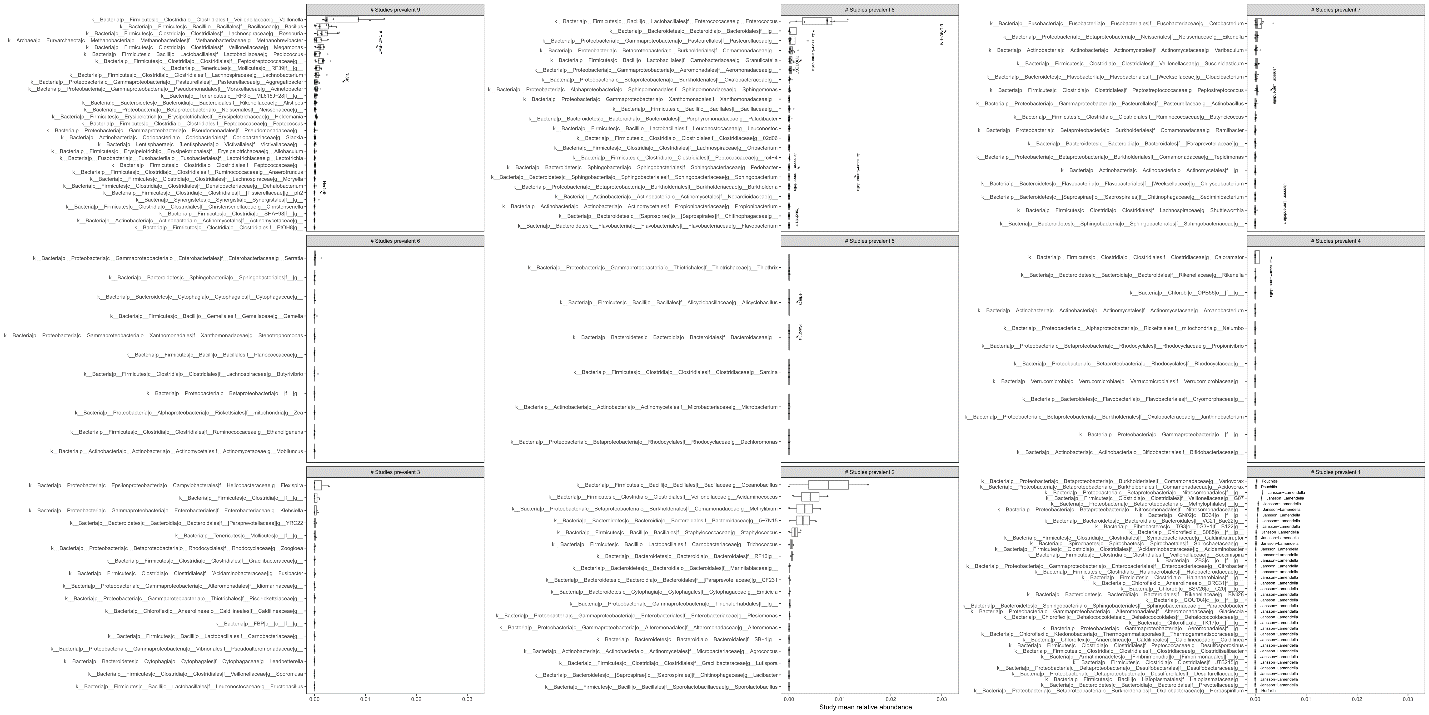


**Fig. S3: Per-study prevalence of taxa that are not globally present, i.e., missing from only one or a few studies.** Features are grouped by number of present studies.


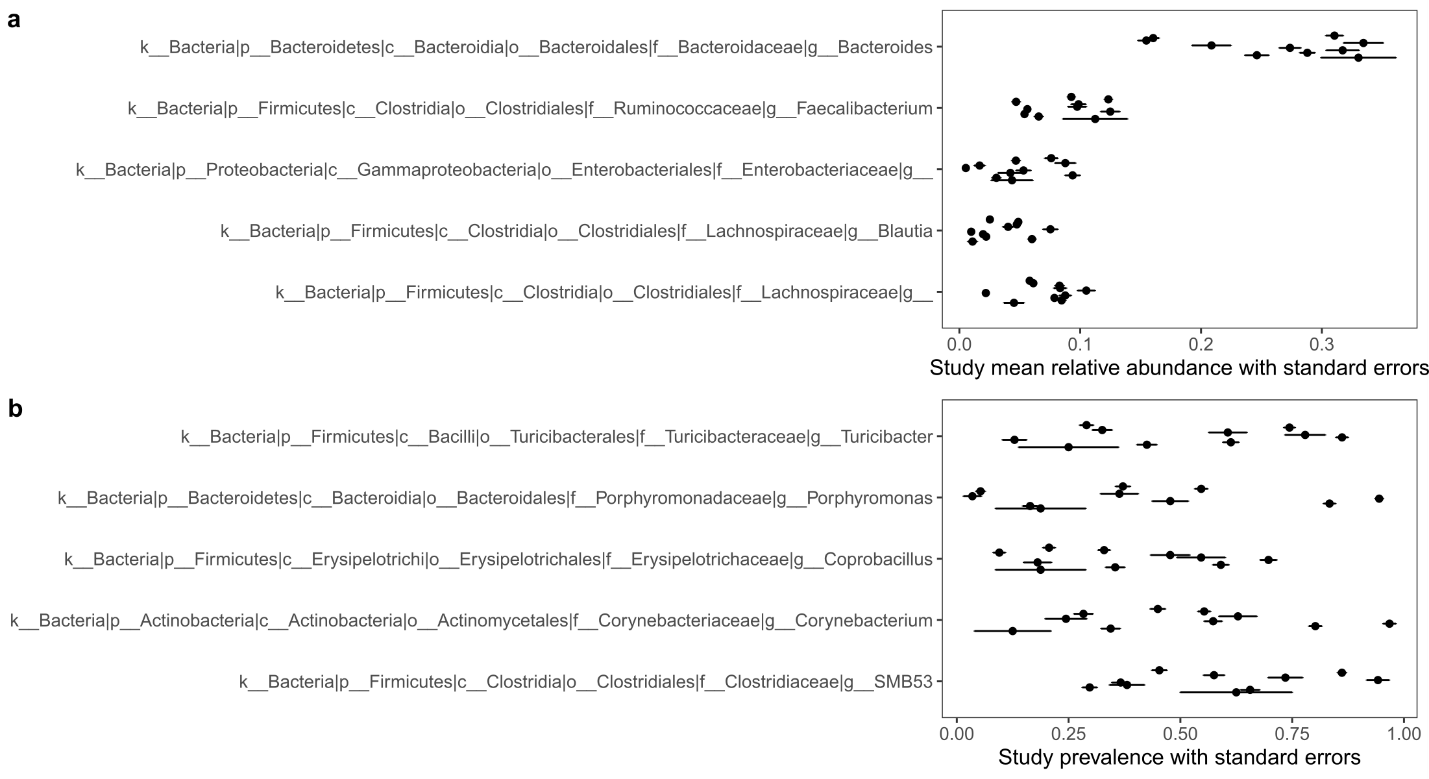


**Fig. S4: Observed differences in per-study mean abundance and prevalence exceed those expected by chance.** We examined the top features in **Fig. S2** for the standard error in their per-study mean abundance and prevalence to ensure that the observed study differences are not due to chance. **a)** The spread across studies of per-study mean abundances far exceeds those expected from their standard errors. As in **Fig. S2a**, each point represents a study-specific mean abundance of one feature, with bars indicating its standard error. **b)** The spread across studies of per-study prevalence also exceeds that expected from their standard errors. Each point represents a study-specific prevalence of one feature, with bars indicating standard errors.


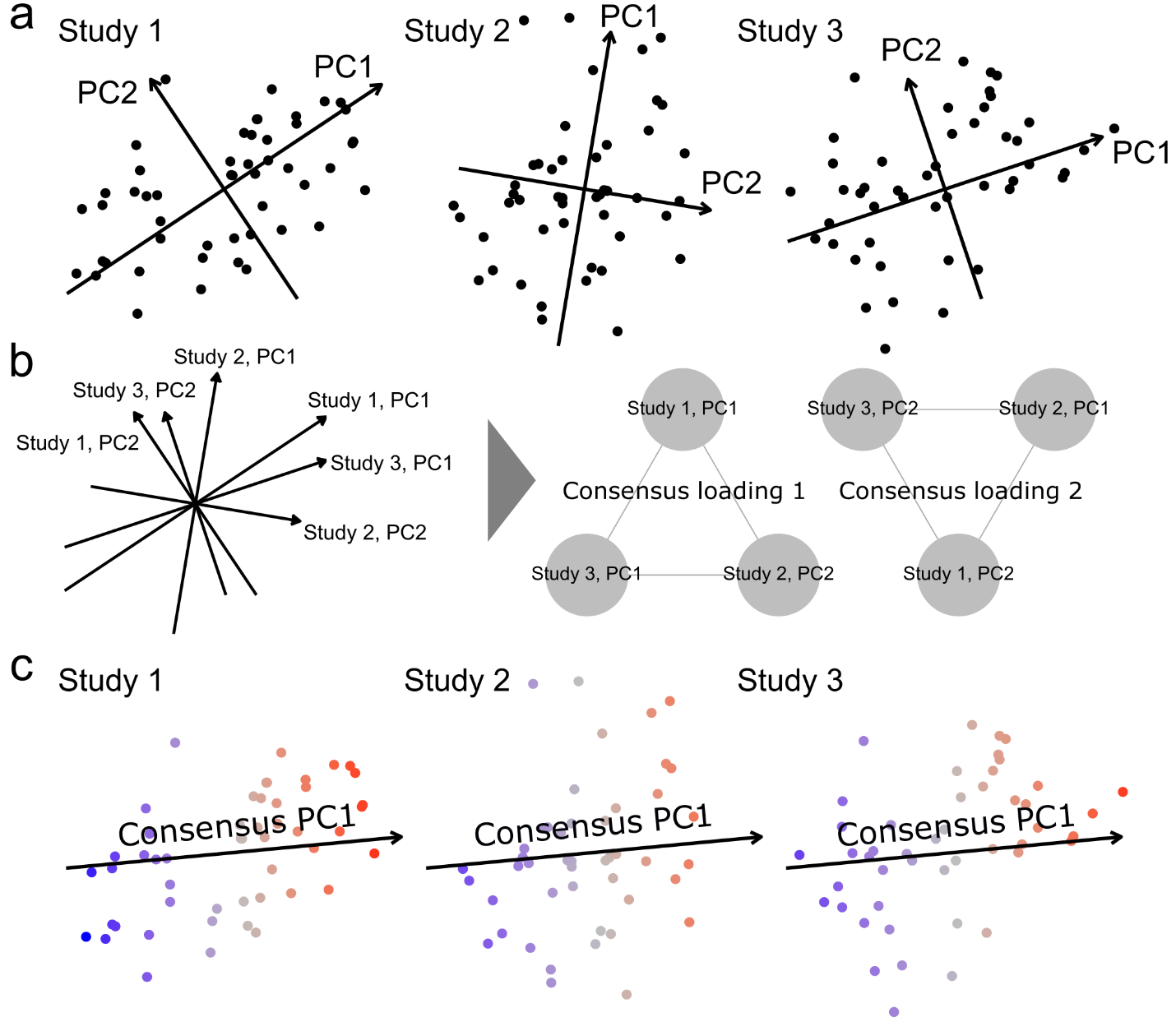


**Fig. S5: MMUPHin_Continuous constructs correlated principal component loadings for robust continuous structure discovery. a)** Principal component analysis is performed in individual studies, identifying the strongest signals present in each, before **b)** principal component loadings from different studies are compared to each other, identifying loadings that are highly similar to each other. **c)** Clusters of similar PC loadings are identified through network community detection, representing strong, recurrent, continuous variation patterns across multiple studies, which can then be used to assign robust, continuously varying gradients in individual cohorts (**d**).


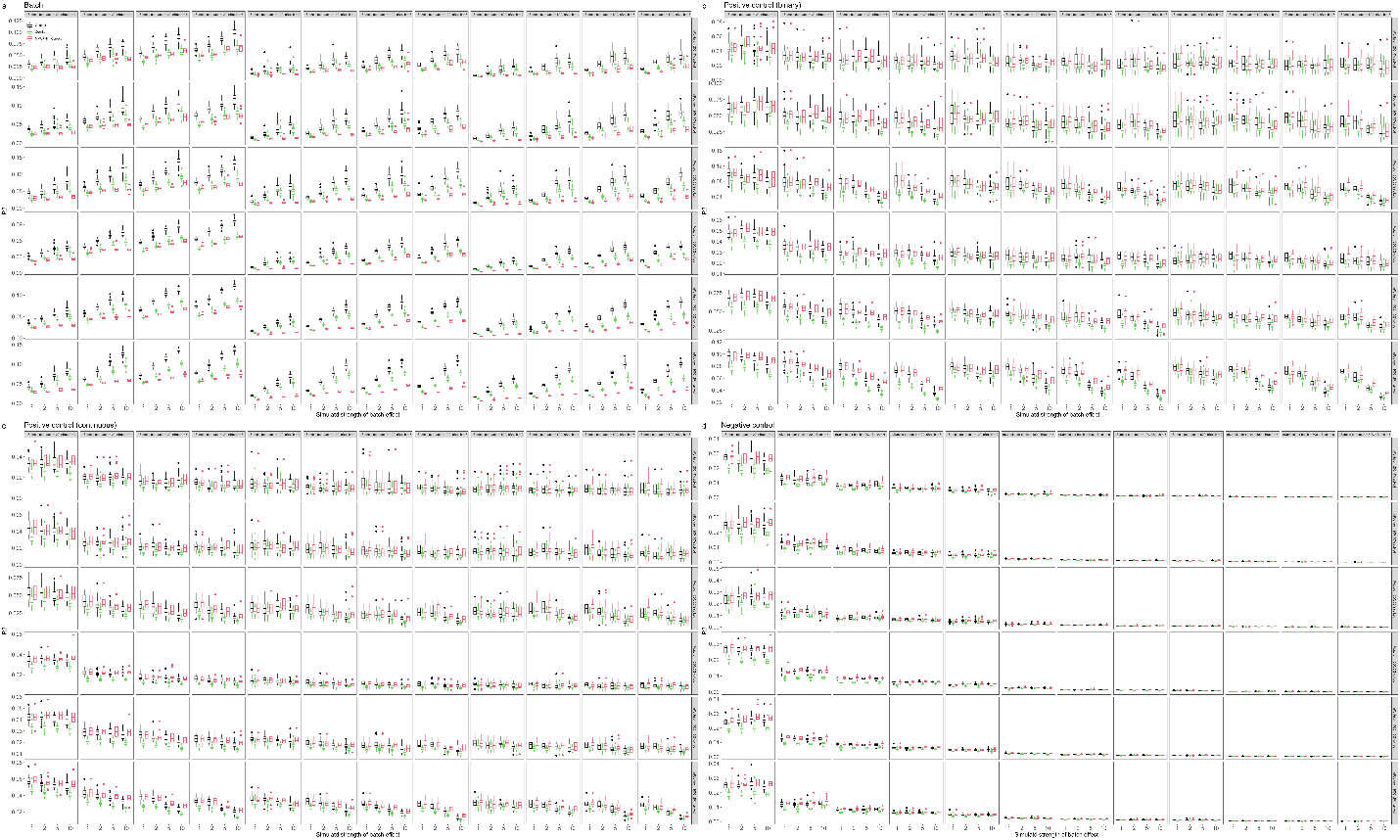


**Fig. S6: Full set of performance evaluation and comparison of MMUPHin's batch adjustment method (MMUPHin_Correct). a-d:** Panels are organized by variables (batch, binary positive control, continuous positive control, and negative control) evaluated by the PERMANOVA R2. For **a-d**, the panel at second row, rightmost column corresponds most closely with our collection of real-world studies for meta-analysis, in terms of data characteristics : eight batches and four thousand samples in total versus ten real studies and 4,789 samples post filtering; 200 microbial features versus 249 real genera; 10% spiked features at batch effect size 10 that yielded ~10% PERMANOVA R2 for batch effect and 3% R2 for binary exposure, versus 10.98% for studies and 3.48% for sample type observed in real data.


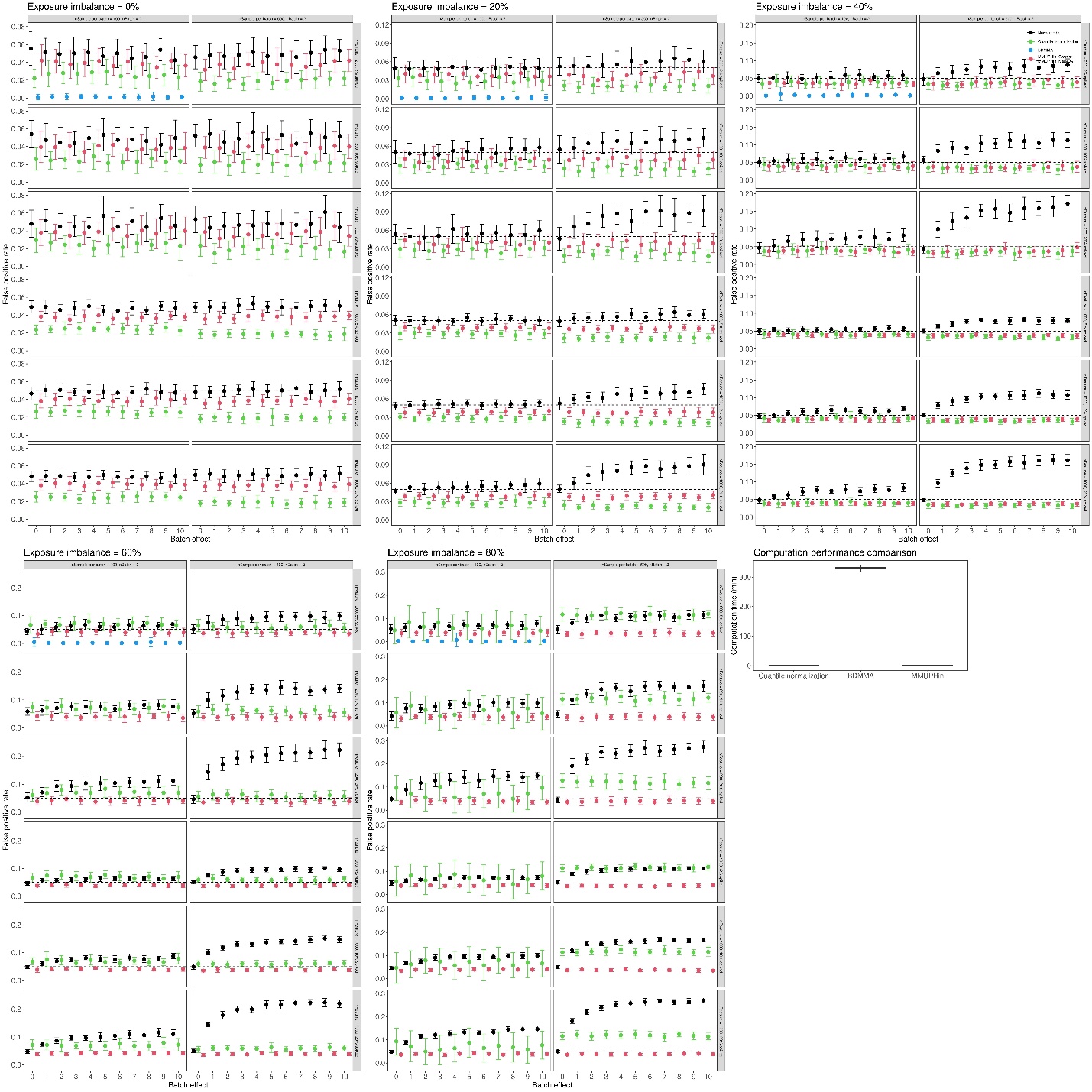


**Fig. S7: Full set of performance evaluation and comparison of MMUPHin's meta-analysis differential abundance testing method (MMUPHin_MetaDA).** MMUPHin_Correct and MMUPHin_MetaDA consistently controls false positive rates across different confounding cohort exposure distribution imbalance set up, when compared to naïve regression, quantile normalization, and BDMMA methods (**a-e**). Note that due to computational cost, BDMMA (purple) was only evaluated for the subset of simulation cases most similar to those evaluated in its publication[19].


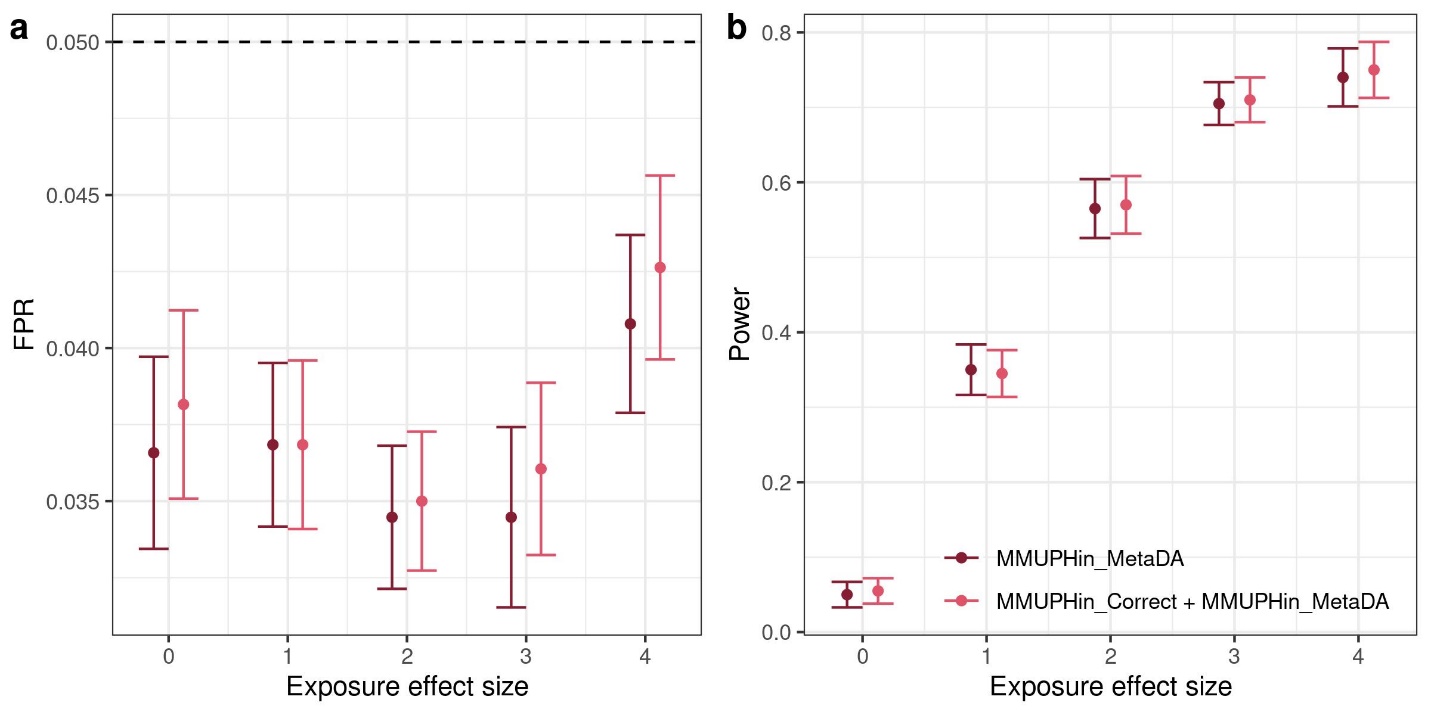


**Fig. S8: Full set of performance evaluation and comparison of MMUPHin's discrete structure discovery method, MMUPHin_Discrete. a-d** Panels are organized by simulated true number of clusters (3-6).

**
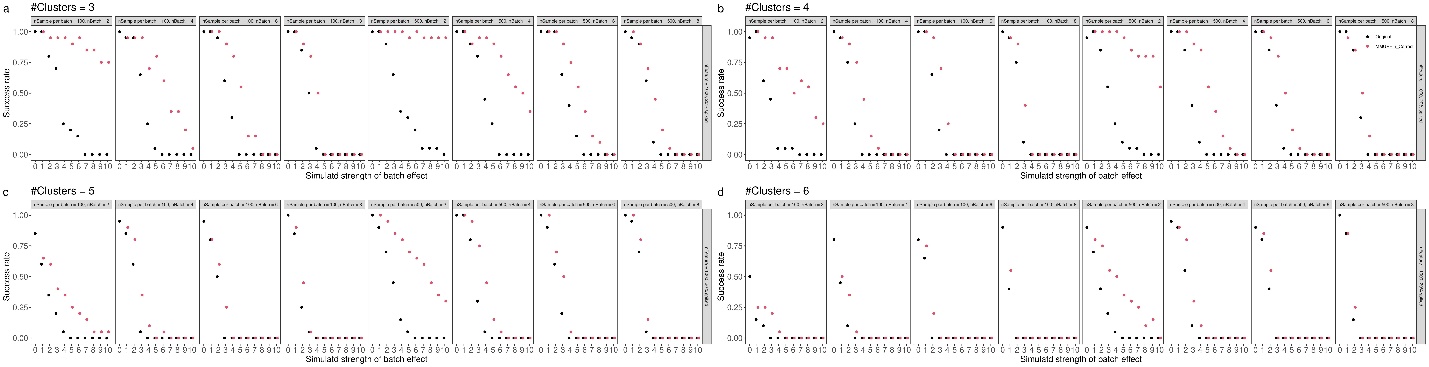
**

**Fig. S9: Full set of performance evaluation and comparison of MMUPHin's discrete structure discovery method, MMUPHin_Discrete. a-d** Panels are organized by simulated true number of clusters (3-6).


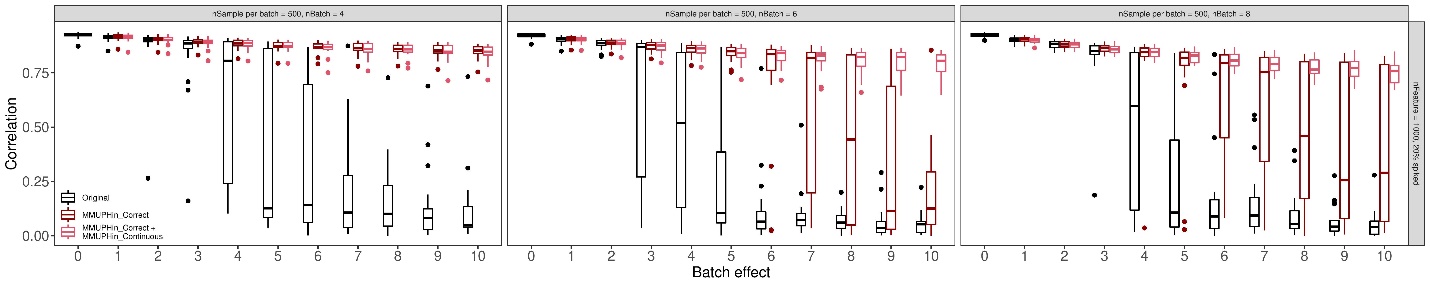


**Fig. S10: Full set of performance evaluation and comparison of MMUPHin's continuous structure discovery method, MMUPHin_Continuous.**

**
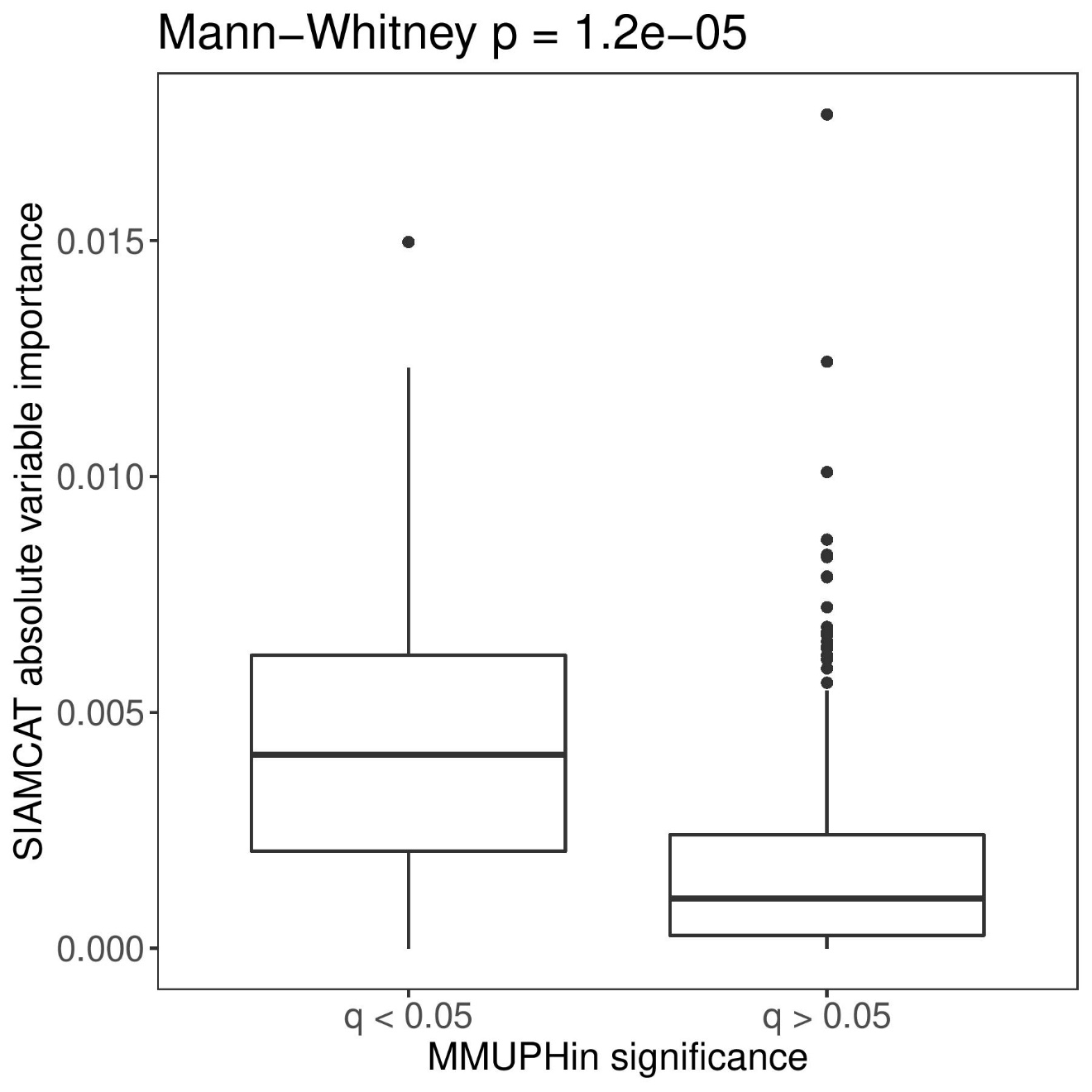
**

**Fig. S11: MMUPHin_MetaDA meta-analysis findings agree with SIAMCAT machine learning feature importances.** We trained SIAMCAT machine learning models predicting IBD status in each individual study where both IBD and control samples were available. From the per-study trained models, each genus’s median relative importances were averaged. These were then compared against genera significance levels (q < 0.05) as identified by MMUPHin. MMUPHin significant features had higher absolute average variable importance as reported by SIAMCAT (p=1.2e-5, one-sided Mann-Whitney U test).


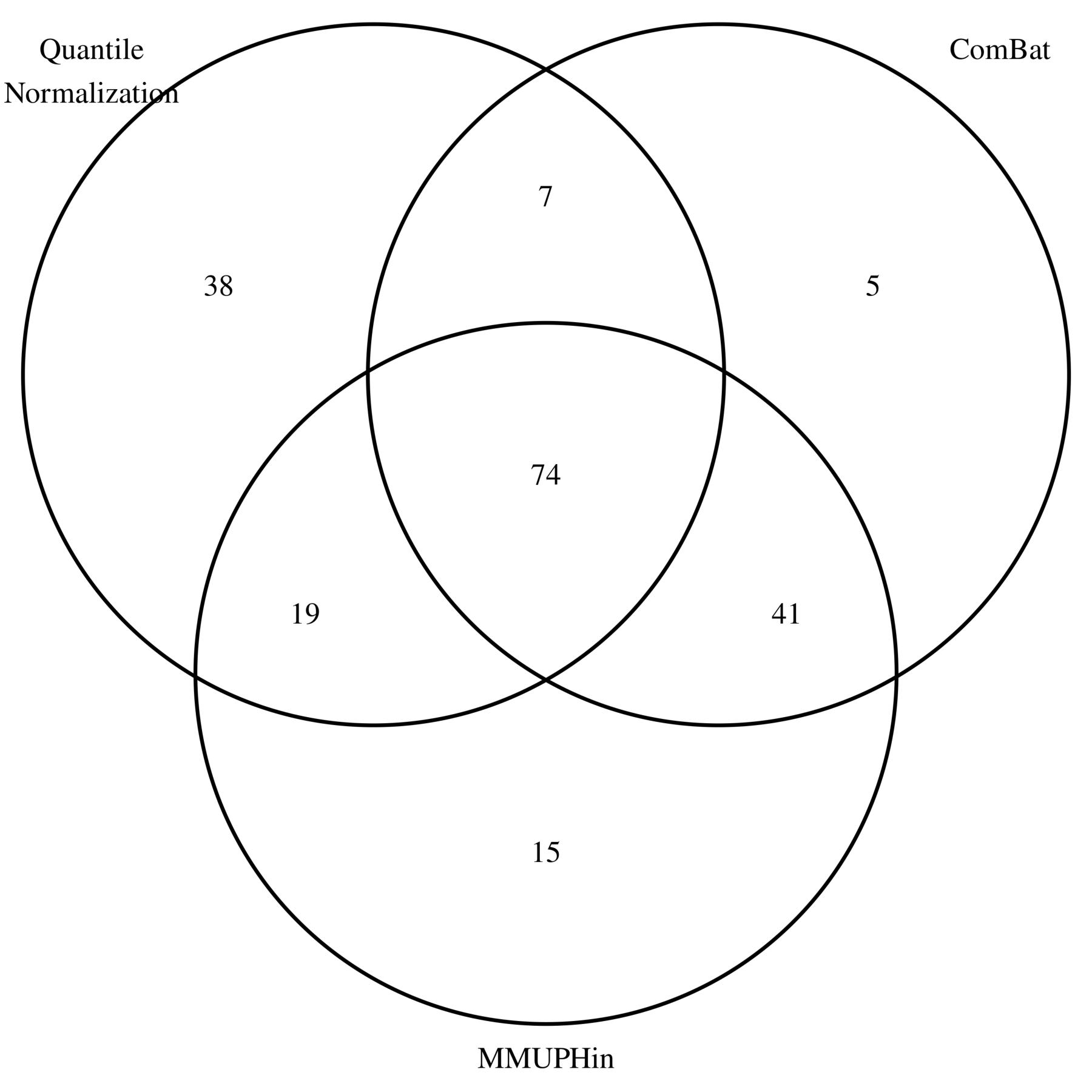


**Fig. S12: MMUPHin_Correct batch correction outperforms quantile normalization and ComBat in real-world IBD association analysis.** Venn diagram indicates the number of features identified individually or jointly after batch correction by quantile normalization, ComBat, or MMUPHin_Correct when comparing IBD versus control microbial profiles. For quantile normalization, univariate nonparametric Mann-Whitney tests were performed for the normalized percentiles pooled across studies, as recommended in [18]. For ComBat and MMUPHin_Correct corrected microbial relative abundances, univariate two-sample t-tests were performed for consistency with the quantile normalization analysis. MMUPHin_Correct correction identified the greatest number of significant genera (p < 0.05) out of the three approaches. Additionally, it showed the largest agreement with both of its alternatives.


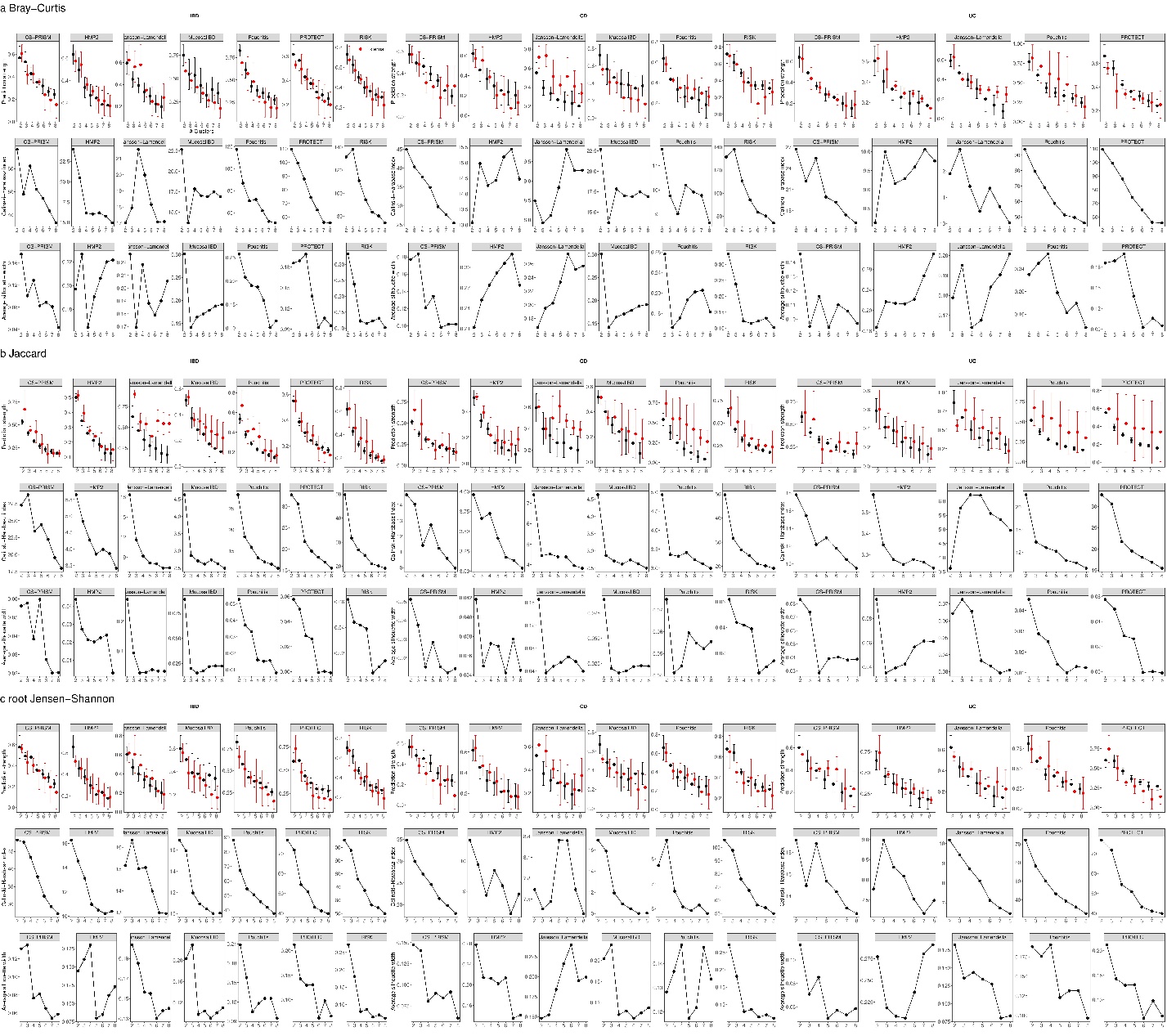


**Fig. S13: Comprehensive evaluation provided no evidence to support discrete enterotypes in IBD cohorts.** Combinations of clustering strength evaluation metrics (prediction strength, Calinski-Harabasz index, and average silhouette width), paired with different dissimilarity measures (**a** Bray-Curtis, **b** Jaccard, and **c** square root Jensen-Shannon divergence) were evaluated, with no consistent support for the existence of enterotypes (i.e., “peaking” of clustering strength metric at a particular cluster number $k$) across cohorts.


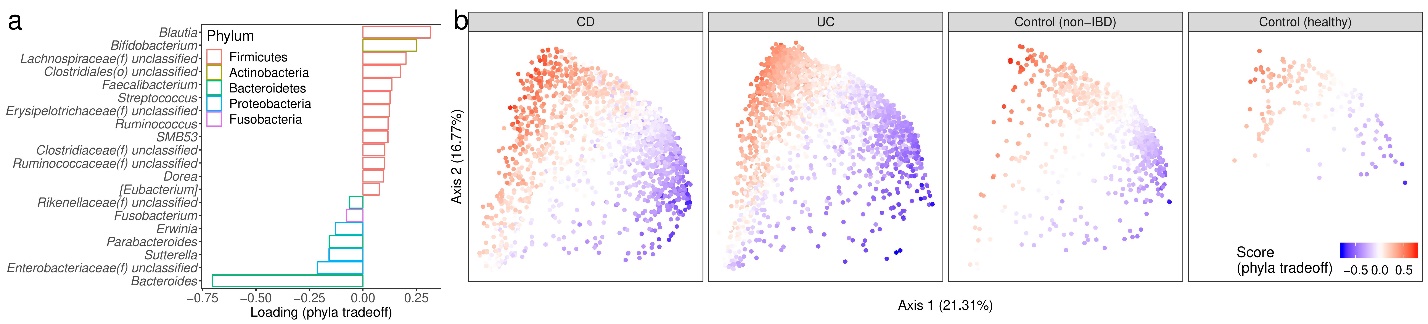


**Fig. S14: Second continuous score identified by MMUPHin_Continuous characterizes dominant phyla trade-off in the IBD gut. a)** Consensus loading vector corresponding to the second continuous score is dominated, in opposing directions, by genera from the Firmicutes and Bacteroidetes phyla. **b)** The phyla trade-off score is consistently present across diseased and control populations.


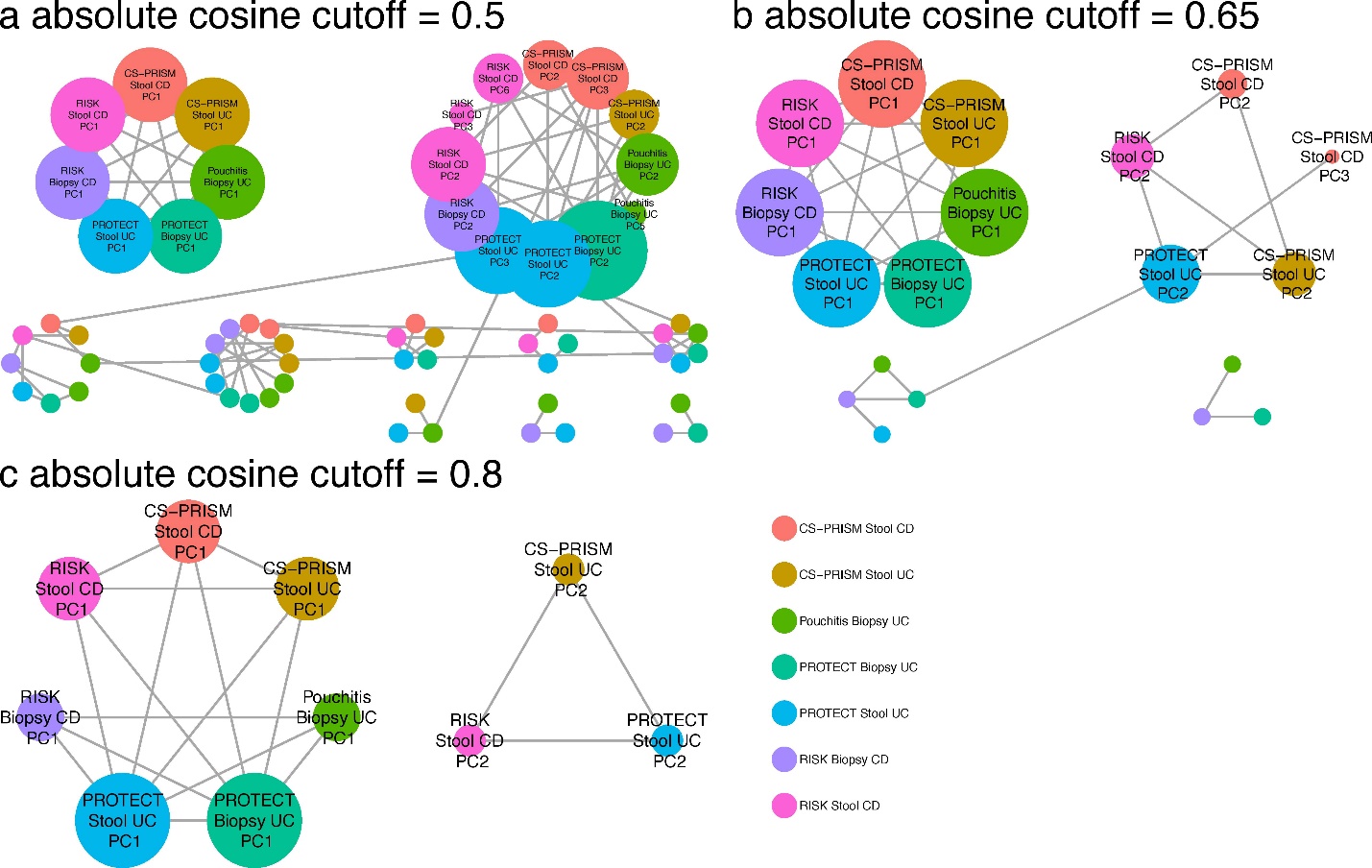


**Fig. S15: Continuous PC loading clusters are not sensitive to cosine coefficient cutoff.** The same core members of each of the two PC loading clusters are repeatedly observed across different cutoffs (**a-c**).


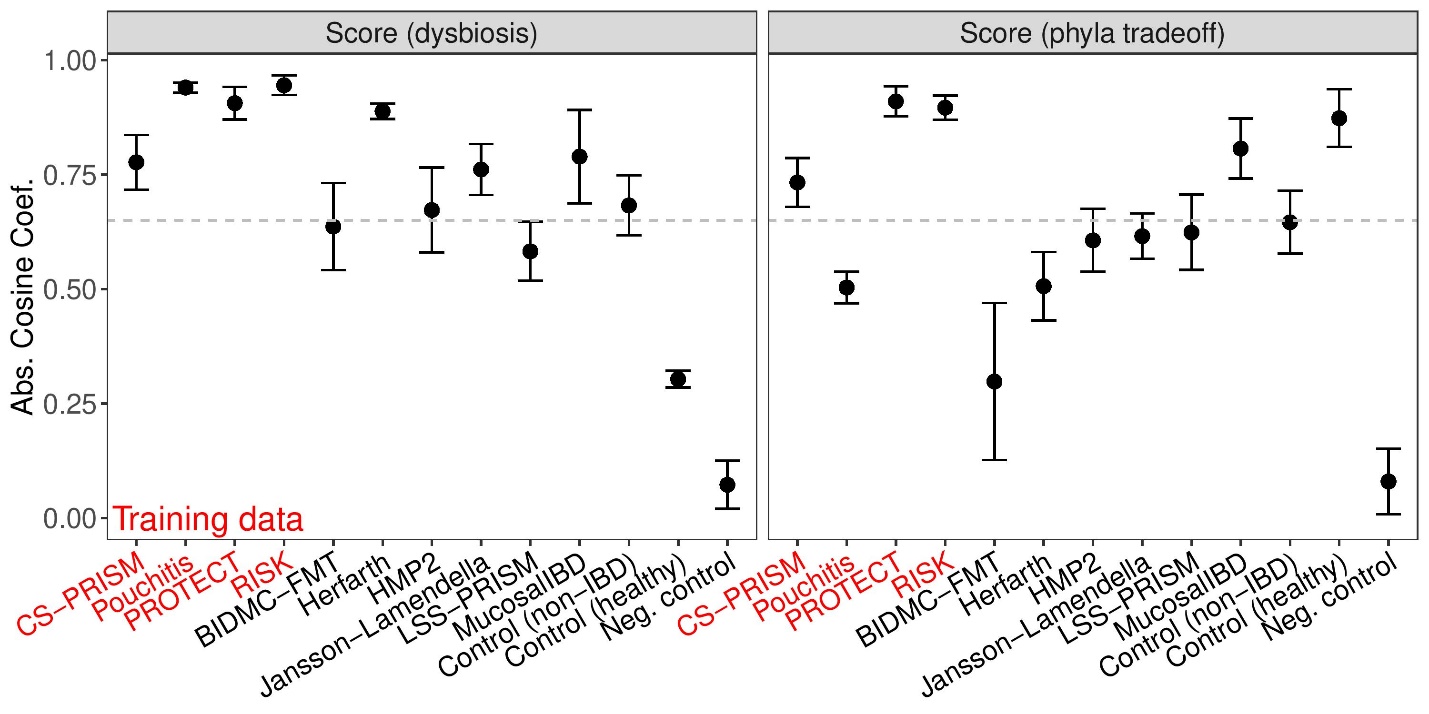


**Fig. S16: The two continuous PC scores are validated in testing cohorts.** The dysbiosis score was reproduced in all training and validation cohorts except for in healthy populations and the negative control dataset (**a**), while the common gut phyla trade-off score was reproduced across most of the diseased and healthy populations except for negative control (**b**).


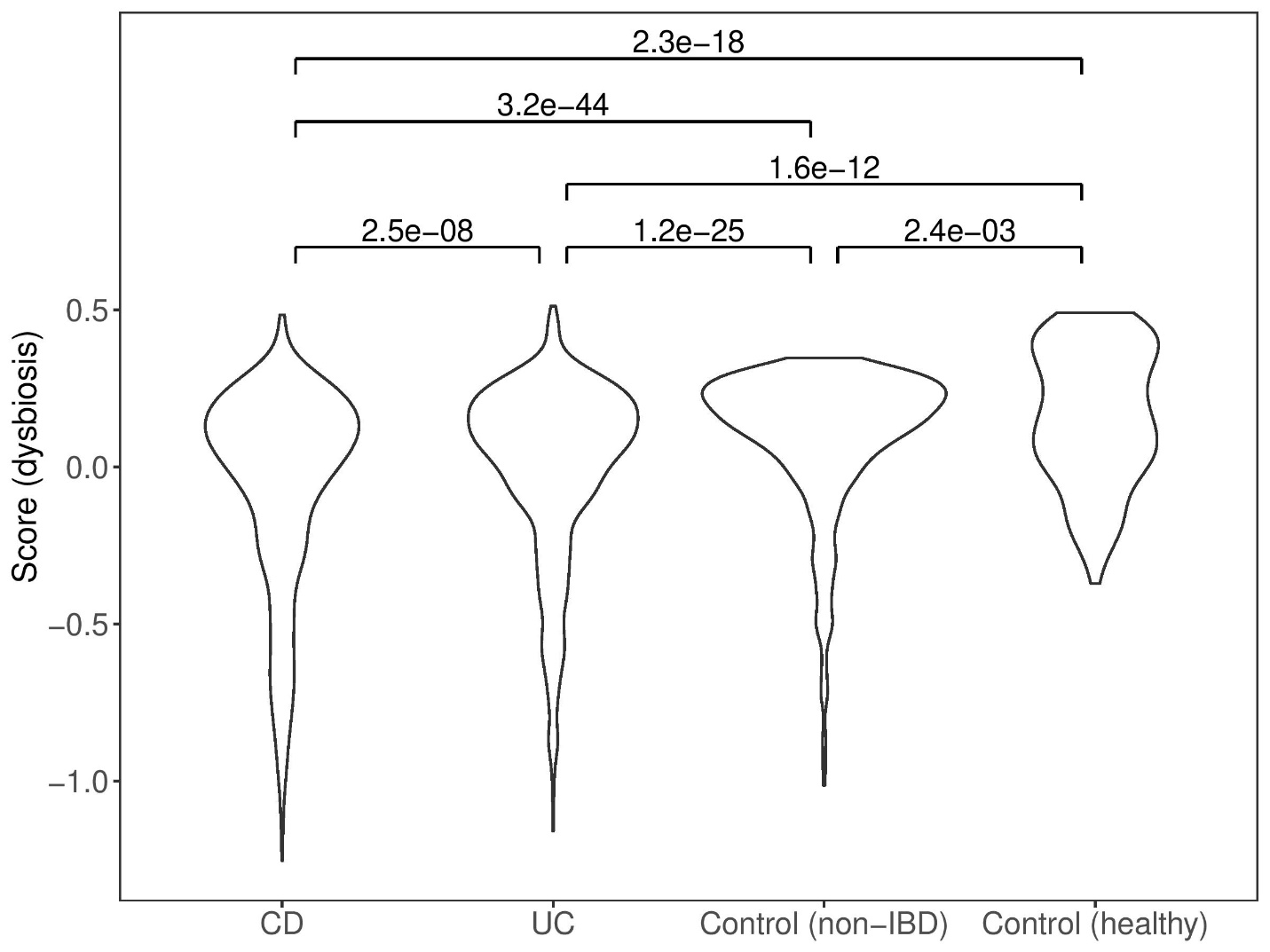


**Fig. S17: The dysbiosis score differentiates between CD, UC, non-IBD control, and healthy populations.** P-value was obtained via two-sample Wilcoxon rank sum tests and adjusted by Benjamini-Hochberg procedure.


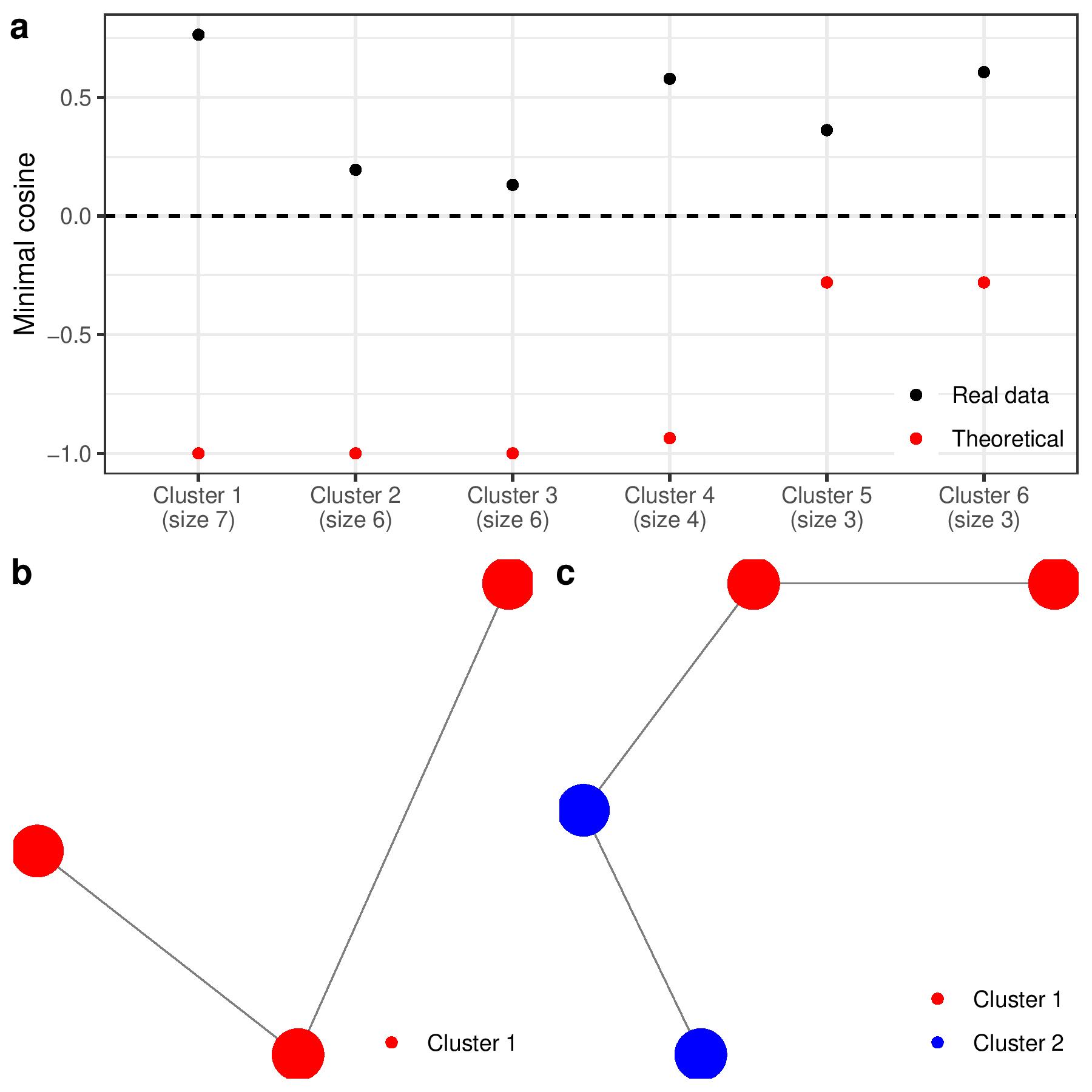


**Fig. S18 Empirical evidence and methodological properties that facilitate consistent PC cluster discoveries in MMUPHin_Continuous.** Consistent PC clusters are defined as ones where all PCs have positive cosine coefficients, after necessary sign corrections are performed (**Methods**). **a)** Empirical evidence that clustered PCs tend to be consistent. With a cosine threshold of 0.6, all identified PC clusters are consistent with minimal cosines above zero, and much higher than their theoretical smallest values. Theoretical lowest cosines for each cluster are determined by cluster sizes and the cosine cutoff used to construct the graph (details in **Additional File 2: Supplemental Notes**). This provides evidence that real-world data with recurrent biological signals are much better behaved than theoretical worst-case scenarios. **b-c)** Modularity-based network clustering avoids long “chain” clusters which tend to be inconsistent. Our adopted clustering algorithm maximizes the modularity score, which balances between bigger clusters, and smaller, better-connected ones (**Additional File 2: Supplemental Notes**). Consequently, long “chains” that are poorly connected intracluster such as in **c** are broken into separate, better-connected ones. This naturally avoids inconsistent clusters, for which poor connectivity is a necessary condition by definition.


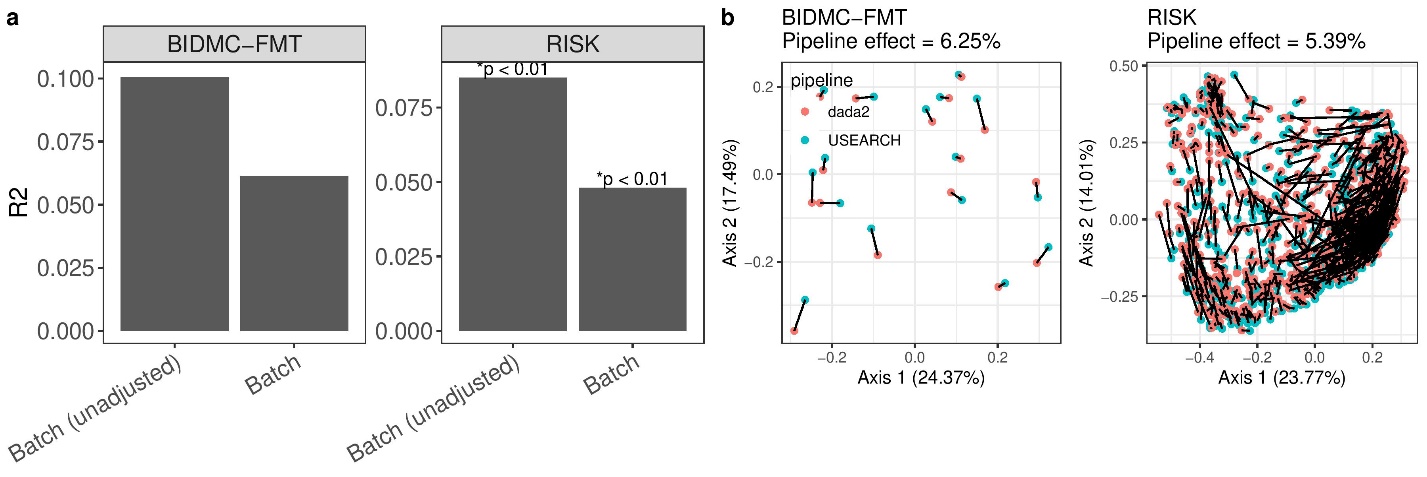


**Fig. S19: MMUPHin meta-analysis method and results apply comparably to OTU and ASV abundance profiles.** **a)** MMUPHin_Correct batch correction successfully reduces batch difference in ASV abundance profiles, in both small and large sample size studies with different numbers of technical batches. Batch effects in either study are quantified through PERMANOVA R2 as in Figure 3a. **b)** When aggregated at the genus level, the choice of OTU- versus ASV-based bioinformatics pipelines has limited impact on the generated abundance profiles, given properly configured and quality-controlled OTU formation parameters (as previously described[66]). Pipeline effect is again quantified through PERMANOVA R2 statistics.
